# Clinical prediction of prognostic outcome of intravenous thrombolysis in mild stroke

**DOI:** 10.1097/MD.0000000000042848

**Published:** 2025-06-20

**Authors:** Chao Feng, Bing Dai, Mo Yang, Feifei Yu, Qing Liang, Yinglei Li, Tao Qie

**Affiliations:** a Department of Emergency Medicine, Baoding No.1 Central Hospital, Baoding, China.

**Keywords:** intravenous thrombolysis, LASSO, mild ischemic stroke, nomogram, prognosis

## Abstract

Development and validation of an easy-to-visualize nomogram for acute mild ischemic stroke (MIS) with unfavorable outcome 3 months after application of alteplase intravenous thrombolysis. A retrospective cohort study analysis was conducted at the Baoding First Central Hospital, involving 461 patients diagnosed with acute MIS who received alteplase thrombolysis within the treatment time window. The LASSO regression technique was employed to identify significant variables and develop nomograms. The model’s performance was assessed through area under curve–receiver operating characteristic curves, calibration plots, and decision curves, followed by a final evaluation of its validity. Five predictors that were found to be significantly associated with a 3-month adverse prognosis in patients who underwent intravenous thrombolysis for mild stroke were identified as door-to-needle time, homocysteine levels, brain natriuretic peptide, postthrombolysis National Institutes of Health Stroke Scale (NIHSS) score (i.e., immediate postthrombolysis NIHSS score, postthrombolysis NIHSS score (P-NIHSS)), and the monocyte to high-density lipoprotein cholesterol ratio. A visualization chart was constructed. The model had strong predictive performance, with an area under curve–receiver operating characteristic curves of 0.904 (95% confidence interval, 0.858–0.944) for the training cohort and 0.852 (95% confidence interval, 0.768–0.917) for the validation cohort. This straightforward predictive model efficiently identifies factors linked to unfavorable prognosis at the 3-month mark following intravenous thrombolytic therapy for acute MIS, thereby enhancing clinical practice and optimizing the distribution of social resources.

## 1. Introduction

Stroke is widely recognized as the second most common cause of mortality and the third most common cause of disability globally, with a significant increase in its global burden.^[1]^ Over half of stroke cases present as minor upon admission, with minor ischemic strokes (National Institute of Health Stroke Scale [NIHSS] ≤ 5) comprising around 30% of all cases. Of these, approximately 30% experience a lack of independent ambulation upon discharge and exhibit functional disability at the 90-day mark.^[2,3]^ Current guidelines recommend intravenous thrombolysis with recombinant tissue plasminogen activator (rtPA) as the primary treatment for acute ischemic stroke (AIS) within 4.5 hours of symptom onset.^[4]^

However, due to the subjective assessment of mildly disabling strokes and the associated risk of hemorrhage, only 13.5% of patients with mild ischemic strokes (MIS) receive intravenous rtPA in clinical practice.^[5]^ In developed nations like the United States, the utilization rate of thrombolysis for minor strokes is <50%,^[6]^ while in underdeveloped countries such as China, this rate is even lower. Research suggests that the effectiveness of intravenous rtPA may be influenced by the initial NIHSS score in cases of moderate to severe strokes, though this correlation is not well understood in patients with minor ischemic strokes (MIS).

The objective of this research was to develop a visual nomogram utilizing the most recent LASSO regression analysis to predict adverse outcomes 3 months post-thrombolysis in patients with mild stroke. This retrospective study was conducted at a specialized stroke center with the goal of early identification and intervention to minimize the disability associated with mild stroke, enhance the effectiveness of thrombolysis in MIS, and optimize the allocation of clinical and social resources.

## 2. Materials and methods

### 2.1. Research subjects

Our data were obtained from Baoding First Central Hospital in a retrospective study from January 2017 to February 2023. This study was in accordance with the principles established in the Helsinki Declaration and had been approved by the Ethics Committee of our institution (ethical batch number: 2023-017). This was a retrospective study, no patient consent was required due to the approval of the hospital’s ethical review body committee, and all data were anonymized to ensure confidentiality. The inclusion criteria for this study are as follows: participants must be at least 18 years of age, have a baseline NIHSS score of 5 or less, have experienced an AIS within 4.5 hours of onset, meet the criteria for intravenous thrombolysis, have been treated with a standard dose of alteplase (0.9 mg/kg),^[7]^ and have provided informed consent from both the patient and their family. The exclusion criteria for this study include: individuals with a history of stroke and a modified Rankin scale (mRS) score of 1 or higher prior to the current episode, those who underwent endovascular therapy, individuals who died within 24 hours of receiving thrombolysis, patients diagnosed with stroke-like symptoms after hospital admission, individuals with incomplete test data, and patients who missed scheduled follow-up visits.

### 2.2. Variables of interest

Baseline demographic characteristics (including age and sex) and vascular risk factors (including history of hypertension, diabetes mellitus, coronary artery disease, and atrial fibrillation) were validated by a trained neurologist. The baseline NIHSS score (i.e. the NIHSS score ahead thrombolysis, ANIHSS), post-thrombolysis NIHSS score (i.e. the NIHSS score immediately after thrombolysis, PNIHSS) were assessed by a trained neurologist. Blood samples were collected from patients within 24 hours of admission. Laboratory data included baseline blood glucose levels, white blood cell count, hemoglobin (HB) count, platelets count, neutrophils count, lymphocytes count, monocytes count, eosinophils count, ratios of neutrophils to lymphocytes, platelets to neutrophils, monocytes to neutrophils, neutrophils to eosinophils and eosinophils to monocytes, prothrombin time, and partially activated prothrombin time. Other laboratory test results included blood homocysteine (HCY) levels, lactate dehydrogenase, brain natriuretic peptide (BNP), creatinine, uric acid, total cholesterol levels, and triglyceride levels, high-density lipoprotein cholesterol, low-density lipoprotein cholesterol, the ratio of high-density lipoprotein cholesterol and low-density lipoprotein cholesterol and the ratio of monocytes to high-density lipoprotein cholesterol (MHR). Baseline systolic blood pressure, baseline diastolic blood pressure, onset-to-treatment time, and door-to-needle time (DNT) were measured on admission. Missing data were interpolated using multiple interpolation. The etiologic classification of ischemic stroke is defined according to an extended version of the trial of Org 10172 in Acute Stroke Treatment classification.^[8]^

### 2.3. Outcome measures

The patient’s condition was assessed using the 3-month mRS^[9]^ score. Clinical outcomes were categorized as poor prognosis (mRS score of 3–6) or good prognosis (mRS score of 0–2). Evaluation was conducted by a senior physician through telephone callback or face-to-face meeting, with discrepancies resolved by a third physician.

### 2.4. Statistical methods

The data analysis was performed using the R Statistical Software (Version 4.0.2; R Foundation for Statistical Computing, Vienna, Austria). Continuous variables were assessed using the Kolmogorov–Smirnov Goodness of fit test, with normally distributed variables reported as mean ± standard deviation and abnormally distributed variables reported as median and interquartile range. Categorical variables were presented as frequency and percentage (%). Interpolation was employed to address missing values.^[10]^ All data were entered into the R system and randomly divided into training and validation cohort by 7:3.^[11]^ The initial variables underwent screening through LASSO^[12]^ regression to identify pertinent predictors and reduce the risk of model overfitting. Those variables identified through LASSO regression were then incorporated into the subsequent multifactorial Logistic regression analysis. Variables with a significance level of *P* < .05 in the multifactorial logistic regression analysis were deemed statistically significant. We constructed a nomogram using the outcomes of the binary logistic regression model, and then validated the nomogram by using the validation cohort. The performance of the nomogram was evaluated through receiver operating characteristic curve (ROC) analysis, with the area under the curve calculated to assess its performance. The accuracy of the model was determined by calibrating the graph and comparing predicted probabilities with actual results. The clinical utility of the model was assessed through decision curve analysis and probabilistic net benefit analysis. Additionally, a reasonableness analysis of the model was conducted. Statistical significance was defined as *P* < .05.

## 3. Results

### 3.1. Baseline characteristics

**Figure 1.**
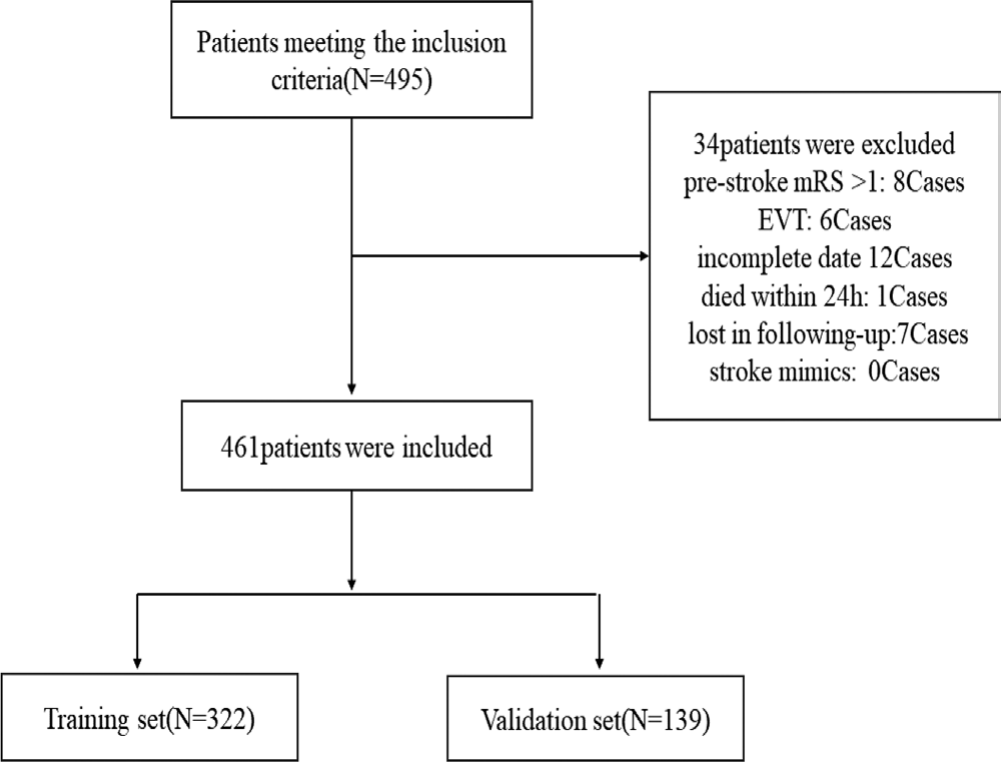
Study flowchart.

Between January 2017 and February 2023, a total of 495 patients met the inclusion criteria for the study. After excluding 34 patients who did not meet the criteria, 461 patients were deemed eligible for data analysis, comprising 322 individuals in the training group and 139 in the validation group (Fig. 1 and Table 1).

**Table 1:** Comparison of baseline information for training and validation sets.

Variables	Total (n = 461)	Validation set (N = 139)	Training set (N = 322)	*P* value
Age, y Median (Q1, Q3)	64 (55, 70)	64 (55.5, 71)	64 (55, 70)	.580
Gender, n (%)				.601
Female	318 (69)	93 (67)	225 (70)	
Male	143 (31)	46 (33)	97 (30)	
BMI, Median (Q1, Q3)	25.06 (22.49, 26.99)	25.61 (22.95, 27.06)	24.49 (22.04, 26.91)	.109
TOAST, n (%)				.527
LAA	235 (51.0)	70 (50.4)	165 (51.2)	
SAO	146 (31.7)	52 (37.4)	94 (29.2)	
CE	75 (16.3)	13 (9.4)	62 (19.3)	
SOE	4 (0.8)	3 (2.1)	1 (0.3)	
SUE	1(0.2)	1(0.7)	0 (0.0)	
SBP, Median (Q1, Q3)	145 (134, 157)	142 (132, 156)	145 (135, 157)	.168
DBP, Median (Q1, Q3)	82 (76, 89)	80 (75, 87)	82 (77, 89)	.04
ANIHSS, Median (Q1, Q3)	2 (2, 4)	2 (2, 4)	3 (2, 4)	.129
Post thrombolysis, NIHSS Median (Q1, Q3)	2 (1, 3)	2 (1, 3)	2 (1, 3)	.37
OTT, min, Median (Q1, Q3)	126 (90, 172)	128 (84, 180)	126 (94, 170)	.906
DNT, min Median (Q1, Q3)	45 (34, 68)	45 (32.5, 67)	46 (35, 69)	.465
Smoking, n (%)				.894
*No*	225 (49)	69 (50)	156 (48)	
*Yes*	236 (51)	70 (50)	166 (52)	
Drinking, n (%)				.760
*No*	295 (64)	87 (63)	208 (65)	
*Yes*	166 (36)	52 (37)	114 (35)	
Diabetes, n (%)				.102
*No*	368 (80)	104 (75)	264 (82)	
*Yes*	93 (20)	35 (25)	58 (18)	
Hypertension, n (%)				.742
*No*	206 (45)	60 (43)	146 (45)	
*Yes*	255 (55)	79 (57)	176 (55)	
Hyperlipidemia, n (%)				.174
*No*	454 (98)	137 (99)	317 (98)	
*Yes*	7 (2)	2 (1)	5 (2)	
Atrial fibrillation, n (%)				.409
*No*	433 (94)	133 (96)	300 (93)	
*Yes*	28 (6)	6 (4)	22 (7)	
HCY, n (%)				.175
*No*	340 (74)	103 (74)	237 (74)	
*Yes*	121 (26)	36 (26)	85 (26)	
Previous stroke, n (%)				.518
*No*	397 (86)	117 (84)	280 (87)	
*Yes*	64 (14)	22 (16)	42 (13)	
CHD, n (%)				.397
*No*	374 (81)	109 (78)	265 (82)	
*Yes*	87 (19)	30 (22)	57 (18)	
WBC counts × 10^9^/L, Median (Q1, Q3)	7.15 (5.79, 8.59)	7.25 (5.8, 8.46)	7.08 (5.79, 8.75)	.908
HB, g/L Mean ± SD	145 (134, 157)	145 (132, 155.5)	145.5 (135, 157)	.315
PLTconut × 10^9^/L, Median (Q1, Q3)	218 (181, 256)	218 (185.5, 250.5)	218 (178, 260.75)	.861
Ncount × 10^9^/L, Median (Q1, Q3)	4.31 (3.35, 5.5)	4.4 (3.49, 5.72)	4.25 (3.3, 5.4)	.3
Mcount × 10^9^/L, Median (Q1, Q3)	0.49 (0.38, 0.62)	0.51 (0.4, 0.62)	0.48 (0.38, 0.61)	.647
Lcount × 10^9^/L, Median (Q1, Q3)	1.93 (1.41, 2.5)	1.75 (1.35, 2.34)	2 (1.49, 2.63)	.017
NLR, ratio Median (Q1, Q3)	2.13 (1.45, 3.39)	2.33 (1.77, 3.78)	2.07 (1.39, 3.32)	.009
MNR, ratio Median (Q1, Q3)	0.11 (0.08, 0.15)	0.11 (0.08, 0.14)	0.11 (0.09, 0.16)	.215
Ecount × 10^9^/L, Median (Q1, Q3)	0.11 (0.05, 0.18)	0.11 (0.05, 0.18)	0.11 (0.06, 0.18)	.737
PNR, ratio Median (Q1, Q3)	47.47 (35.58, 64.69)	47.52 (35.34, 64.2)	47.37 (35.85, 65.01)	.909
NER, ratio Median (Q1, Q3)	46.3 (24.2, 102.6)	46.14 (25.1, 106.34)	46.43 (22.66, 90.77)	.362
EMR, ratio Median (Q1, Q3)	0.2 (0.1, 0.33)	0.19 (0.09, 0.32)	0.21 (0.11, 0.35)	.272
PT, s, Median (Q1, Q3)	10.7 (10.1, 11.4)	10.7 (10.2, 11.3)	10.65 (10.1, 11.4)	.693
APTT, s Median (Q1, Q3)	26.4 (24.6, 28.8)	26.2 (24.85, 28.2)	26.5 (24.6, 29.1)	.411
INR2, Median (Q1, Q3)	0.92 (0.88, 0.97)	0.92 (0.88, 0.97)	0.92 (0.88, 0.97)	.473
GLU, μ mol/L Median (Q1, Q3)	6.78 (5.79, 8.71)	7 (5.76, 9.02)	6.64 (5.85, 8.61)	.437
BNP, p g/ml Median (Q1, Q3)	25.5 (11.9, 76)	26.5 (11.6, 73.8)	25.5 (12, 75.72)	.685
LDH, U/L Median (Q1, Q3)	372 (196, 463)	376 (212.5, 469.8)	370 (194.25, 456.55)	.413
Cr, μ mol/L Median (Q1, Q3)	66.4 (57.89, 78.33)	66.8 (58.94, 77.35)	66.2 (57.41, 79.07)	.843
UA, μ mol/L Median (Q1, Q3)	328.6 (265.95, 393.4)	313.2 (263.02, 379.2)	333.15 (266.95, 397.88)	.274
TC, mmol/L Median (Q1, Q3)	3.51 (3.15, 5.21)	4.18 (3.72, 5.66)	4.32 (3.21, 5.55)	.132
TG, mmol/L Median (Q1, Q3)	1.33 (0.93, 1.81)	1.28 (0.88, 1.83)	1.33 (0.96, 1.81)	.436
HDL, mmol/L Median (Q1, Q3)	1.05 (0.9, 1.26)	1.08 (0.92, 1.28)	1.04 (0.9, 1.24)	.497
LDL, mmol/L Median (Q1, Q3)	2.72 (2.12, 3.34)	2.7 (2.08, 3.41)	2.75 (2.15, 3.3)	.722
HDL/LDL, ratio Median (Q1, Q3)	0.39 (0.31, 0.5)	0.39 (0.32, 0.51)	0.39 (0.31, 0.49)	.563
MHR, ratio Median (Q1, Q3)	0.45 (0.34, 0.64)	0.47 (0.33, 0.65)	0.45 (0.34, 0.63)	.737

APTT = partially activated prothrombin time, BNP = brain natriuretic peptide, CE = cardioembolism, CHD = coronaryheartdisease, DBP = diastolic blood pressure, DNT = door-to-needle time, EMR = the ratio of eosinophils to monocytes, HCY = homocysteine, HDL = high-density lipoprotein cholesterol, INR = international normalized ratio, LAA = large-artery atherosclerosis, LDH = lactic dehydrogenase, LDL = low-density lipoprotein cholesterol, MHR = the ratio of monocytes to high-density lipoprotein cholesterol, MNR = the ratio of monocytes to neutrophils, NER = the ratio of neutrophils to eosinophils, NIHSS = National Institute of Health stroke scale, NLR = the ratio of neutrophil to lymphocyte, OTT = onset-to-treatment time, PLT = platelets, PNR = the ratio of platelet to neutrophil, PT = prothrombin time, SAO = small-artery occlusion, SBP = systolic blood pressure, SOE = other determined etiology, SUE = undetermined etiology, TC = total cholesterol, TG = triglyceride, TOAST = Trial of ORG 10172 in Acute Stroke Treatment, UA = uric acid, WBC = white blood cell.

### 3.2. Variable selection

In this study, 48 potential indicators associated with poor prognosis after intravenous thrombolysis were examined and compared between the 2 groups.

**Figure 2.**
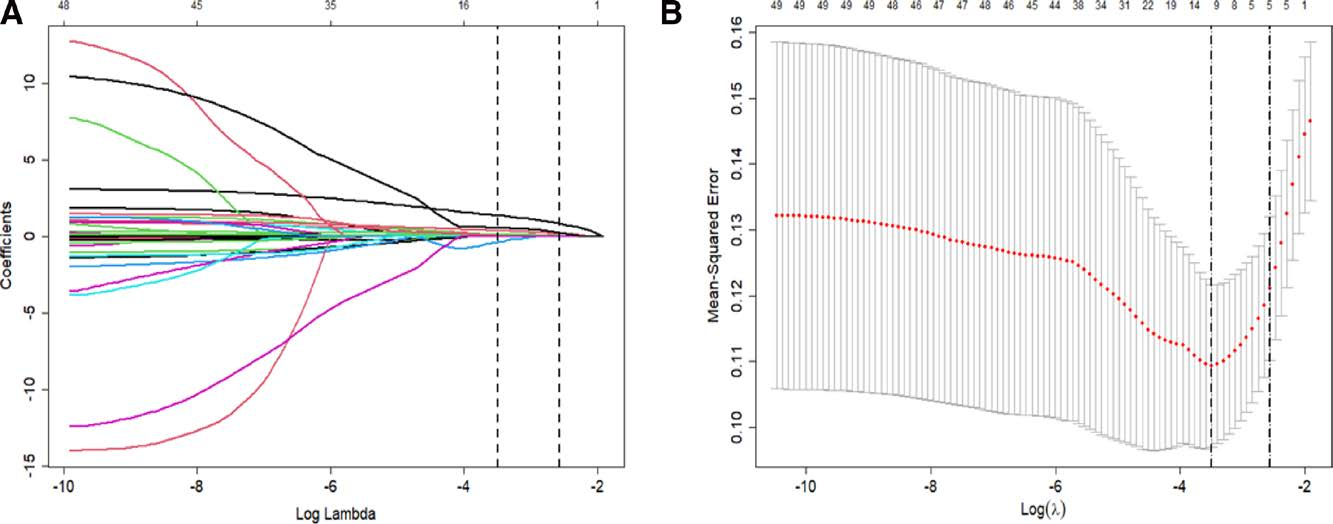
(A) In LASSO regression coefficient profiles, individual lines represent different predictor variables as the regularization parameter (lambda) increases. (B) LASSO regression Lambda Selection: This graph shows the cross-validation curve for model tuning. The lambda value that results in the least cross-validation error is highlighted.

The LASSO regression technique was employed to mitigate overfitting and enhance model accuracy, with the implementation of the glmnet package in R (Fig. 2A, B). Ten-fold cross validations were conducted to identify the optimal regularization parameter (λ = 0.0769738). Penalty coefficient compression was utilized to select 6 key variables, namely DNT, HCY, ANIHSS, PNIHSS, BNP, and MHR (Table 2).

**Table 2:** Coefficients and lambda. 1SE value of the LASSO regression.

Variable. Variable	Variable. Coefficient	Lambda.1SE
DNT	0.001003352	0.0769738
HCY	0.8559737942	
BNP	0.0003244736	
ANIHSS	0.1969975508	
PNIHSS	0.0565311403	
MHR	0.2805130694	

BNP = brain natriuretic peptide, DNT = door-to-needle time, HCY = homocysteine, MHR = the ratio of monocytes to high-density lipoprotein cholesterol.

### 3.3. Multivariate analysis

Incorporating the above 6 variables into a logistic regression resulted in the 5 indicators most associated with poor 3-month prognostic outcomes.

Multivariate regression analysis was subsequently employed to demonstrate that the variable DNT (odds ratio [OR], 1.014; 95% confidence interval [CI], 1.003–1.026; *P* = .008), HCY(OR, 6.441; 95% CI, 3.070–14.01; *P*<.001), BNP (OR, 1.809; 95% CI, 1.381–2.420; *P* = .007), PNIHSS (OR, 1.002; 95% CI, 1.001–1.004; 0.002), and MHR (OR, 4.547; 95% CI, 1.797–15.04; 0.007) served as a significant predictor of unfavorable prognosis(Table 3).

**Table 3:** Independent risk factors associated with 3-month poor outcomes after intravenous thrombolysis in the training cohort.

Variable	B	SE	OR	CI	Z	*P*
DNT	0.015	0.00564	1.014	1.014 (1.003–1.026)	2.636	.008
HCY	1.863	0.38501	6.441	6.441 (3.070–14.01)	4.838	<.001
BNP	0.593	0.14252	1.809	1.809 (1.381–2.420)	4.162	<.001
PNIHSS	0.002	0.00082	1.002	1.002 (1.001–1.004)	3.053	.002
MHR	1.515	0.56352	4.547	4.547 (1.797–15.040)	2.688	.007

BNP = brain natriuretic peptide, CI = confidence interval, DNT = door-to-needle time, HCY = homocysteine, MHR = the ratio of monocytes to high-density lipoprotein cholesterol, OR = odds ratio.

### 3.4. Predictive model development

**Figure 3.**
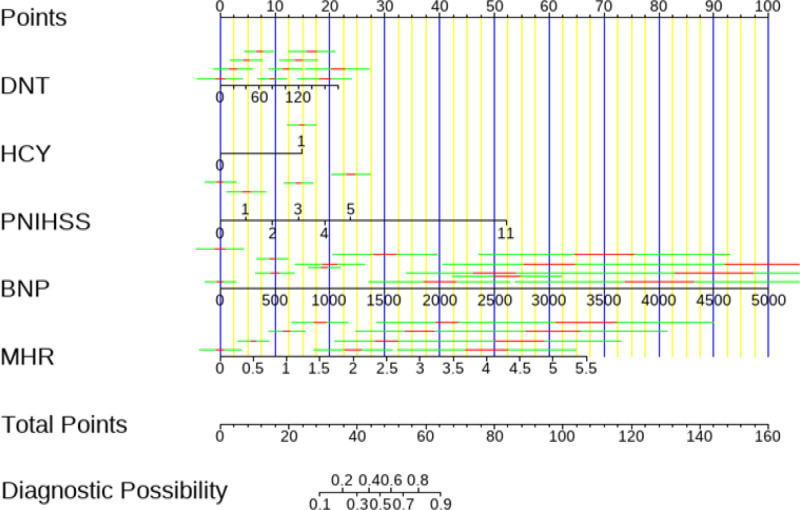
The Nomogram for predicting the 3-month unfavorable outcome in minor ischemic stroke (MIS).

Through a multifactorial logistic regression analysis of patients with AIS, five predictors associated with poor prognosis were identified: DNT, HCY, BNP, PNIHSS, and MHR. Nomograms were developed utilizing these variables, with corresponding point values assigned to each predictor. The summation of these point values facilitated the estimation of the likelihood of a 3-month risk of poor prognosis in AIS patients receiving intravenous thrombolysis (refer to Fig. 3).

### 3.5. Nomogram validation

The model’s discriminatory ability was evaluated by determining the area under curve (AUC)–ROC for the training group (AUC, 0.904; 95% CI, 0.858–0.944); external validation was conducted on the test group with an AUC–ROC of 0.852 (95% CI, 0.768–0.917).

**Figure 4.**
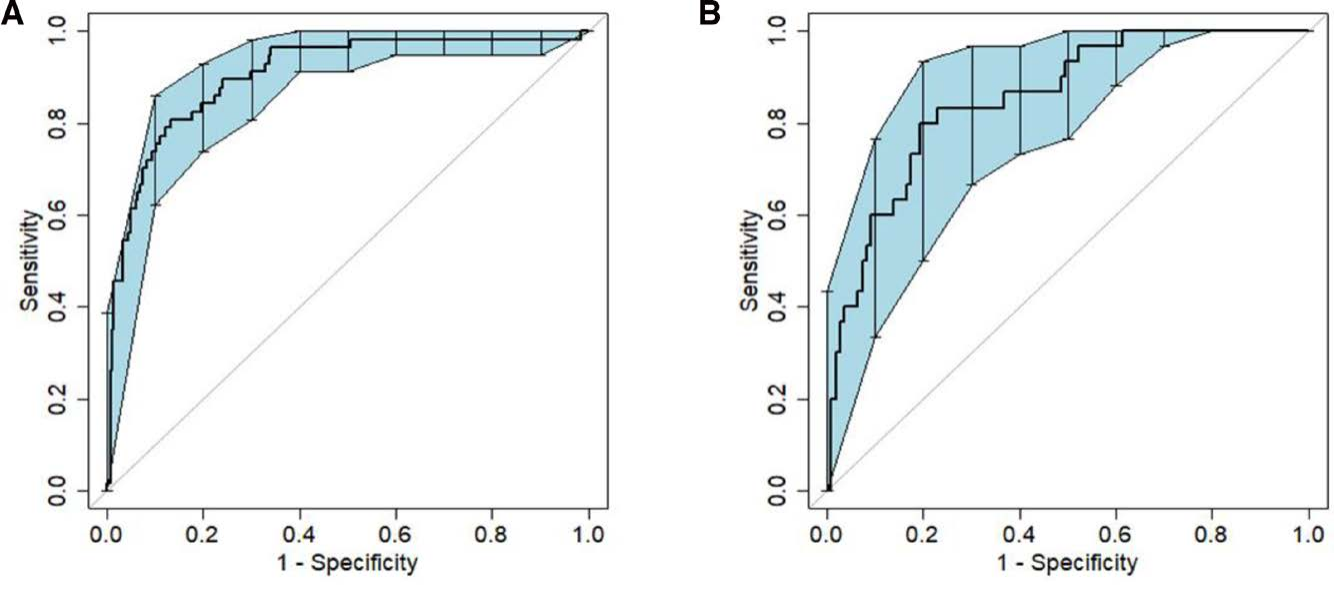
Receiver operating characteristic curves (ROC) of the predictive model for the training dataset (A) and validation dataset (B).

After validating the nomogram, it was found that high levels of sensitivity and specificity were present in both the training and validation datasets. This suggests that the model can effectively distinguish between true positive and true negative cases with poor prognoses. Specifically, in the training set, a cutoff of 0.213 yielded a sensitivity of 80.7% and specificity of 86.3%, while in the validation set, a cutoff of 0.115 resulted in a sensitivity of 80.7% and specificity of 80.0% (Fig. 4A, B).

**Figure 5.**
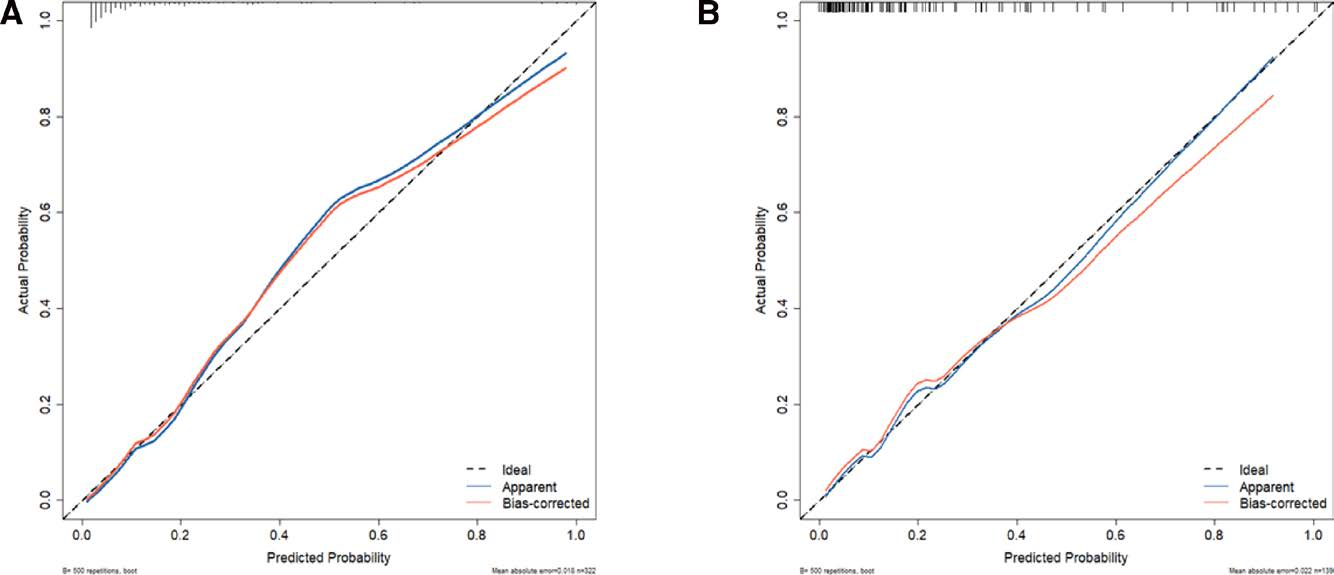
Calibration plot of the nomogram in the training cohorts (A) and validation cohorts (B).

The calibration curves of the predictive model fit well with the ideal curves in the modeling and validation cohorts, showing good fit between the training and validation nomogram calibration curves. It was consistent with the actual probability that the predictive model predicted a poor prognosis (Fig. 5A, B).

**Figure 6.**
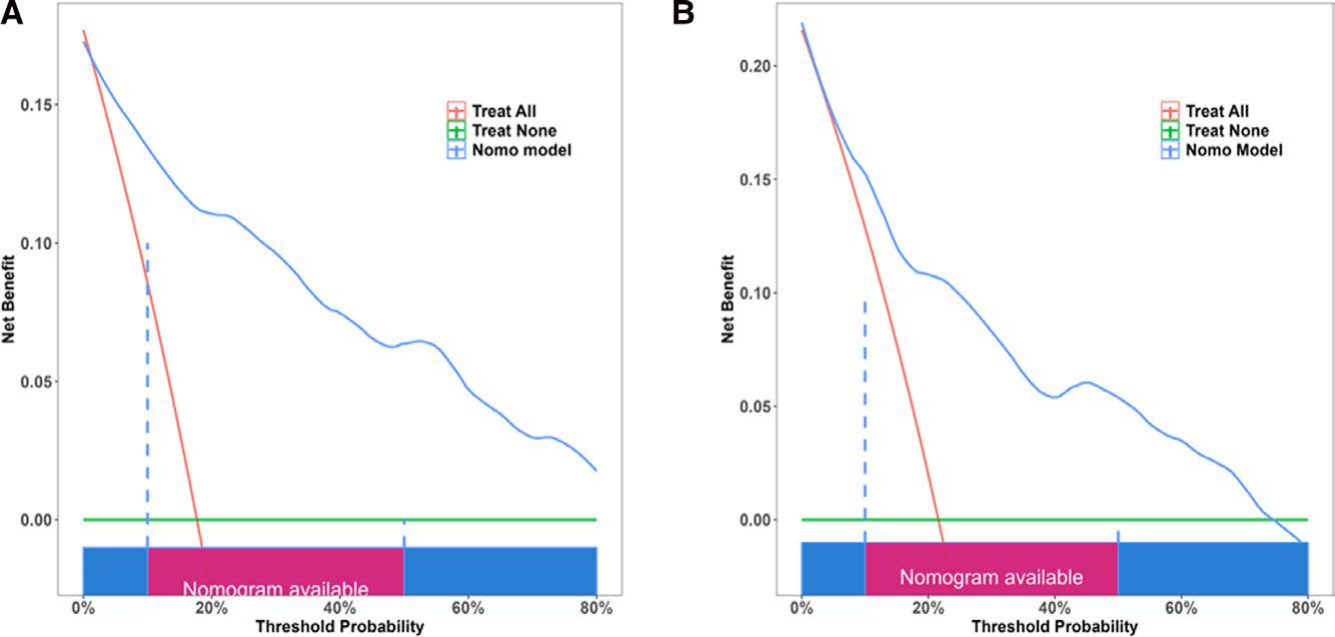
Nomogram decision curve analysis for the training cohort (A) and validation cohort (B).

A decision curve analysis was performed to assess the prognostic value of the established prediction model. The analysis quantified net benefit through the comparison of true-positive and false-positive outcomes, demonstrating that the current nomogram yielded a higher net benefit (Fig. 6A, B).

**Figure 7.**
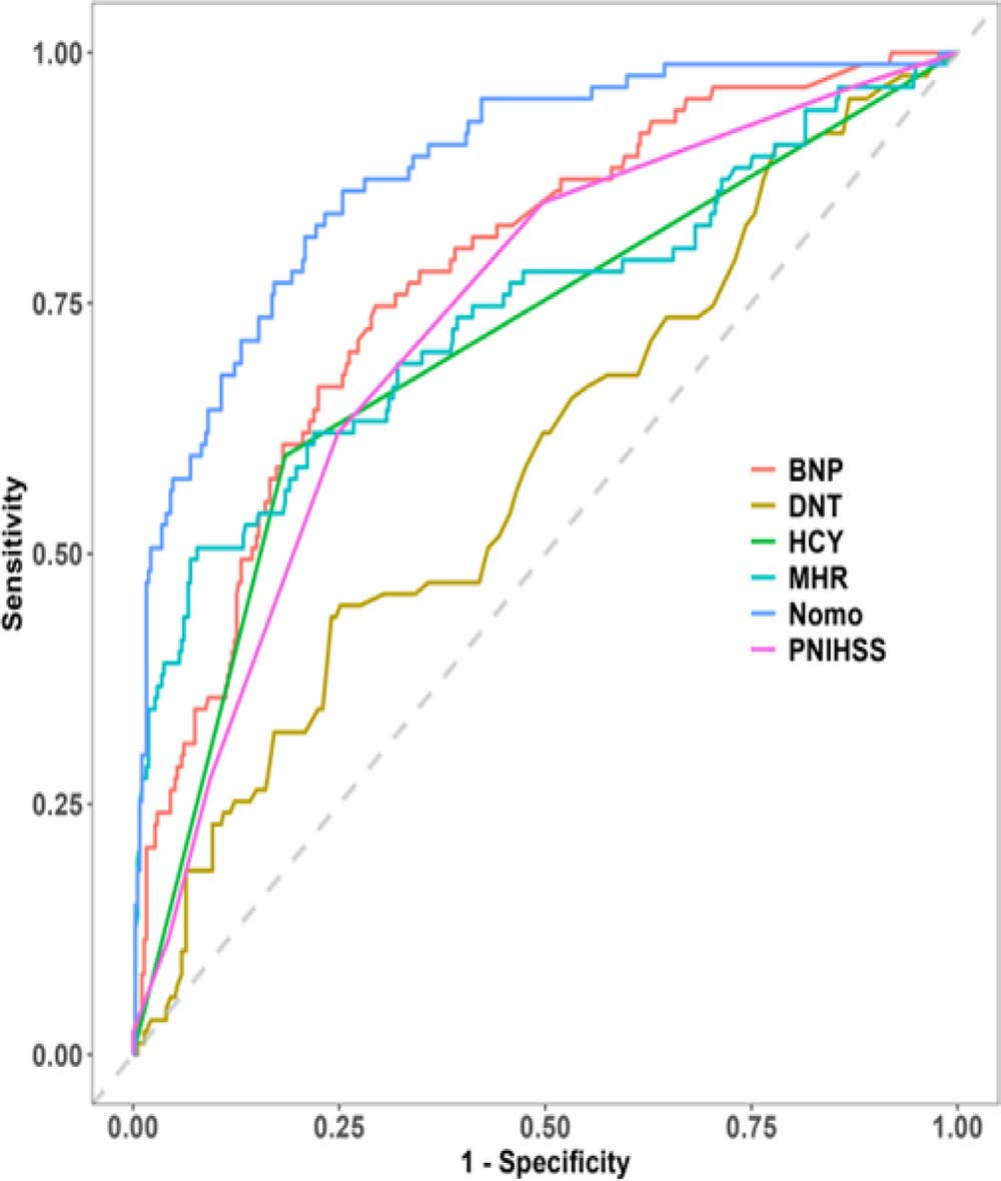
A reasonableness analysis was made based on model probabilities.

Following this, a validation of the model’s rationality was performed, showing that the ROC of the 495-based nomogram demonstrated a statistically significant improvement in comparison to the single-variable nomogram, suggesting a clear rationality (Fig. 7).

**Figure 8.**
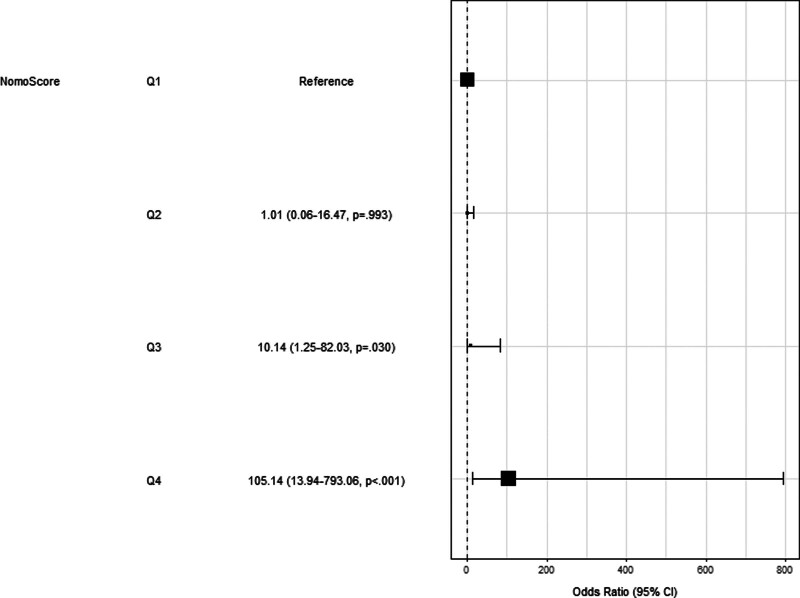
Forest plots based on constructed nomograms predicting unfavorable outcomes 3 months after thrombolysis in mild ischemic stroke.

Furthermore, we have constructed a forest map based on the constructed nomogram, which is seen to have good applicability (Fig. 8).

## 4. Discussion

This research project constructed and verified a concise prognostic boxplot for forecasting the prognosis of mild stroke patients following a 3-month treatment of thrombolytic therapy with alteplase. The graphical representation considers five variables: DNT, HCY levels, BNP levels, the NIHSS score post-thrombolytic therapy, and the mean heart rate (MHR), all of which are readily accessible and cost-effective clinical data commonly utilized in clinical settings.

The NIHSS^[13]^ is a significant independent predictor that reflects the extent of neurological impairment and serves as a crucial prognostic indicator for individuals suffering from ischemic stroke. Various research studies have illustrated the utility of the NIHSS score in evaluating neurological deficits in thrombolyzed patients post 3 months.^[2,14]^ The findings of this study corroborate that in individuals with MIS, the post-thrombolysis NIHSS score is a predictive factor for determining the likelihood of an unfavorable outcome at 3 months. BNP^[15,16]^ was initially discovered in porcine brain extracts and later identified as a cardiac natriuretic hormone. The main trigger for pro-BNP synthesis and release by cardiomyocytes is myocyte stretch. Following secretion, the pre-peptide undergoes cleavage to form biologically active BNP and the residual hormone N-terminal pro-BNP (NT-pro-BNP). Pro-BNP^[17]^ is consistently secreted, with the mature peptide having minimal intracellular reserves. Elevated levels of NT-pro-BNP^[18]^ were associated with an increased likelihood of experiencing adverse outcomes, such as death and vascular events, and poor functional recovery 1 year following the onset of a stroke. This indicates that NT-pro-BNP could serve as a valuable prognostic indicator for ischemic stroke.^[19]^ A study identified a correlation between NT-pro-BNP levels in stroke patients receiving intravenous thrombolysis and adverse 3-month functional outcomes and mortality in individuals undergoing intravenous thrombolysis for stroke. This research represents^[20]^ a novel discovery indicating that BNP levels are linked to unfavorable prognostic outcomes at the 3-month mark following mild ischemic IV thrombolysis stroke. AIS is treated by intravenous thrombolysis, and the shorter the duration from DNT,^[21]^ the greater the benefit and better the prognosis. HCY^[22]^ is a sulfur-containing amino acid that undergoes catabolism requiring the presence of vitamin B₆. Disruption in folate and B-vitamin levels can hinder the conversion of HCY. Research indicates that^[23]^ elevated levels of HCY are an independent risk factor for cardiovascular and cerebrovascular diseases, impacting vascular health. Higher levels of HCY are associated with poorer clinical outcomes in patients with AIS following thrombolytic therapy. The monocyte-to-high-density lipoprotein cholesterol ratio (MHR)^[24]^ has emerged as a novel systemic marker of inflammation linked to atherosclerosis, with the proinflammatory characteristics of monocytes and the protective attributes of high-density lipoprotein cholesterol holding promise for prognostic implications in cardiovascular disease. Recent findings have indicated that the MHR^[25]^ may serve as a prognostic tool in individuals undergoing intravenous thrombolysis for acute stroke.

The effectiveness of rtPA intravenous thrombolytic therapy for mild stroke is a topic of debate. This study introduces an analysis of the efficacy of thrombolysis in patients with mild stroke to determine the probability of a poor 3-month prognosis in patients with MIS thrombolysis. The results indicate that^[25]^ for nondisabling MIS, optimal medical therapy is not significantly different from intravenous thrombolysis. Therefore, conservative medical therapy may be the preferred treatment for patients with nondisabling MIS. Half of patients with a NIHSS score of 0 to 6 exhibit infarction despite receiving thrombolytic therapy, while 40% experience a poor prognosis due to glucose metabolism and hemorrhagic complications.^[26]^ Thus, personalized evaluation is essential for patients eligible for thrombolysis with mild symptoms. Given the variability in outcomes for mild stroke patients undergoing thrombolysis, there is a pressing need for enhanced prognostic tools to predict unfavorable outcomes promptly and accurately, facilitating the application of precision medicine.

This study is subject to several limitations. Firstly, while the data was collected from advanced stroke centers, it is important to note that this study is based on a single-center database. Future research should aim to replicate and confirm these findings through multicenter studies. Secondly, certain variables that are typically associated with the prognosis of AIS, such as hypertension,^[27]^ were not included in the analysis. Lastly, the retrospective nature of this study may introduce biases, including information bias and follow-up bias, which should be addressed in future prospective studies.

## 5. Conclusion

This study developed and validated a nomogram using LASSO regression to predict unfavorable prognosis in patients with acute MIS who have undergone 3 months of intravenous thrombolysis with alteplase. The nomogram incorporates variables such as DNT, HCY, BNP, PNIHSS, and MHR, providing a reliable, simple, and intuitive tool for early assessment of patient prognosis. Implementation of this predictive tool may facilitate timely interventions, reduce disability associated with mild strokes, enhance thrombolytic benefits, and optimize allocation of healthcare resources on a global scale.

## Acknowledgments

The authors thank the staﬀ of the Department of Emergency, the Baoding First Central Hospital, China.

## Author contributions

**Data curation:** Feifei Yu, Qing Liang.

**Supervision:** Feifei Yu, Qing Liang.

**Writing – original draft:** Bing Dai, Mo Yang.

**Writing – review & editing:** Chao Feng, Yinglei Li, Tao Qie.
